# Impact of the Local Inflammatory Environment on Mucosal Vitamin D Metabolism and Signaling in Chronic Inflammatory Lung Diseases

**DOI:** 10.3389/fimmu.2020.01433

**Published:** 2020-07-10

**Authors:** Jasmijn A. Schrumpf, Anne M. van der Does, Pieter S. Hiemstra

**Affiliations:** Department of Pulmonology, Leiden University Medical Center, Leiden, Netherlands

**Keywords:** inflammation, airway mucosa, vitamin D, vitamin D metabolism, host defense, COPD exacerbations

## Abstract

Vitamin D plays an active role in the modulation of innate and adaptive immune responses as well as in the protection against respiratory pathogens. Evidence for this immunomodulatory and protective role is derived from observational studies showing an association between vitamin D deficiency, chronic airway diseases and respiratory infections, and is supported by a range of experimental studies using cell culture and animal models. Furthermore, recent intervention studies have now shown that vitamin D supplementation reduces exacerbation rates in vitamin D-deficient patients with chronic obstructive pulmonary disease (COPD) or asthma and decreases the incidence of acute respiratory tract infections. The active vitamin D metabolite, 1,25-dihydroxy-vitamin D (1,25(OH)_2_D), is known to contribute to the integrity of the mucosal barrier, promote killing of pathogens (via the induction of antimicrobial peptides), and to modulate inflammation and immune responses. These mechanisms may partly explain its protective role against infections and exacerbations in COPD and asthma patients. The respiratory mucosa is an important site of local 1,25(OH)_2_D synthesis, degradation and signaling, a process that can be affected by exposure to inflammatory mediators. As a consequence, mucosal inflammation and other disease-associated factors, as observed in e.g., COPD and asthma, may modulate the protective actions of 1,25(OH)_2_D. Here, we discuss the potential consequences of various disease-associated processes such as inflammation and exposure to pathogens and inhaled toxicants on vitamin D metabolism and local responses to 1,25(OH)_2_D in both immune- and epithelial cells. We furthermore discuss potential consequences of disturbed local levels of 25(OH)D and 1,25(OH)_2_D for chronic lung diseases. Additional insight into the relationship between disease-associated mechanisms and local effects of 1,25(OH)_2_D is expected to contribute to the design of future strategies aimed at improving local levels of 1,25(OH)_2_D and signaling in chronic inflammatory lung diseases.

## Introduction

Vitamin D is a pleiotropic hormone that is well-known for its role in the regulation of calcium and phosphate homeostasis and bone mineralization. The vitamin D receptor (VDR) acts as the receptor for the active form of vitamin D, i.e., 1,25-dihydroxy-vitamin D [1,25(OH)_2_D], and is expressed in nearly all tissues and cell-types and regulates a large number of genes (~0.8–5% of the total genome) (1, 2). As a result, vitamin D affects many additional processes including cell proliferation and differentiation, apoptosis, DNA repair, ion transport, metabolism, cell adhesion, and oxidative stress responses (1, 3). Vitamin D deficiency [serum 25-hydroxy-vitamin D [25(OH)D] <50 nmol/L; 25(OH)D is the main circulating form of vitamin D and its levels are used to assess vitamin D status in the clinic (4, 5) affects more than 30% of the children and adults worldwide and is a major cause of bone diseases such as rickets and osteoporosis (6). Increasing evidence has indicated that vitamin D deficiency is also associated with various other diseases such as cancer, cardiovascular disease, Alzheimer's disease and muscle myopathy, as well as several immune-related diseases such as type 1 diabetes, multiple sclerosis, inflammatory bowel disease (IBD), psoriasis and chronic inflammatory lung diseases including asthma, cystic fibrosis (CF), and chronic obstructive pulmonary disease (COPD) (6–9).

Several studies have now shown that vitamin D deficiency is prevalent in COPD patients and inversely correlated with lung function and severity of the disease (8–12). It is currently unknown whether vitamin D deficiency is a cause or consequence of COPD, since many COPD patients have low physical activity levels and spend most time indoors (13). There are however studies suggesting that low 25(OH)D levels are associated with development of COPD, based on observed associations between polymorphisms in the vitamin D binding protein (VDBP), 25(OH)D serum levels and COPD severity (8, 10, 11, 14). In addition, one study in mice showed that maternal vitamin D deficiency can impair lung -development, -structure and -function in the offspring and suggests that even before birth, maternal 25(OH)D serum levels are important for a healthy lung development (15). This might be relevant, since associations have been found between lower childhood lung function and development of COPD later in life (16). The link between maternal 25(OH)D status and asthma development is however much clearer, since two recent randomized controlled trials (RCTs) have shown that maternal vitamin D supplementation reduces the risk of childhood asthma/recurrent wheeze (17). This might be explained by the fact that multiple vitamin D-regulated genes are transcriptionally active during alveolar maturation and a number of these genes are differentially expressed in asthma (18). Additionally, this protective effect was linked to the GG-genotype of the 17q21 functional SNP rs12936231, which is associated with lower expression of *ORMDL3* and increased sphingolipid metabolism (19). Moreover, maternal circulating 25(OH)D levels affect the gut microbiota and can therefore indirectly modulate immune responses in the lung via the gut-lung-axis (20). Also later in life, optimal 25(OH)D levels remain crucial for keeping the lungs healthy. For example, Heulens et al. showed that subacute and chronic cigarette smoke (CS) exposure decreased lung function and promoted early signs of emphysema and airway inflammation in vitamin D-deficient mice compared to vitamin D-sufficient animals (21). Similarly in an elastase-induced COPD mouse model, topical administration of vitamin D in the lungs counteracted alveolar damage and improved lung function (22). Yet in humans, it is still unclear whether vitamin D status influences COPD development and disease progression. Taken together, these observations suggest an important role for vitamin D during fetal and childhood lung maturation, and indicate that sufficient 25(OH)D levels might contribute to protection against development of childhood asthma and possibly COPD at older age.

Systemic levels of biologically active 1,25(OH)_2_D are tightly regulated to preserve sufficient levels of calcium (Ca^2+^) and phosphate (${\text{PO}}_{4}{}^{2-}$) for optimal bone mineralization, whereas in mucosal tissues locally produced (autocrine) 1,25(OH)_2_D levels and signaling can be elevated or decreased upon exposure to inflammatory mediators, pathogens or inhaled toxicants (6). This could be important, since the inflamed airway mucosa of patients suffering from chronic inflammatory lung diseases is constantly exposed to these disease-associated factors (8, 23, 24). Impaired local levels of 1,25(OH)_2_D and VDR signaling might have consequences for disease pathogenesis and progression. Dysregulated host defenses as found in patients with chronic inflammatory airway diseases include aberrant immune responses, altered microbiome composition, impaired epithelial barrier function, and aberrant secretion of host defense molecules (25–27). Adequate 1,25(OH)_2_D levels may provide protection against these dysregulated processes by maintaining the integrity of the mucosal barrier and promotion of killing of pathogens (e.g., via the induction of the antimicrobial peptide [AMP] hCAP18/LL-37) and via the modulation of both innate and adaptive immune responses (7, 28, 29).

In this review, we first discuss the effects of these disease-associated factors on local synthesis and availability of 1,25(OH)_2_D and 1,25(OH)_2_D-induced responses in the lung mucosa. In the second part of the review we will describe the mechanistic links between vitamin D deficiency and the pathogenesis of chronic inflammatory lung diseases such as asthma, CF and COPD, and discuss recent evidence related to the protective effects of vitamin D on COPD and on COPD exacerbations.

## Mucosal Vitamin D Metabolism in Health

**Figure 1:**
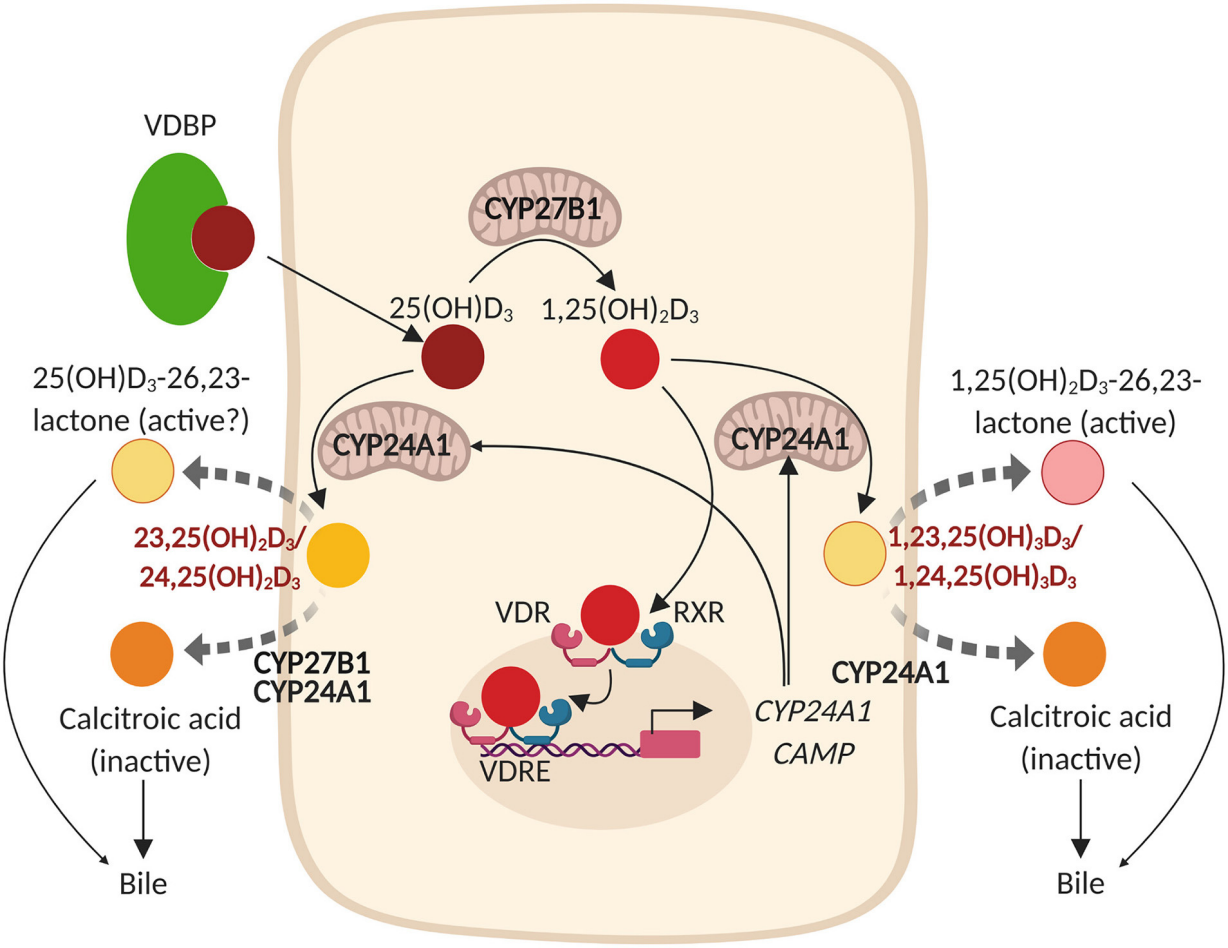
Vitamin D metabolism and expression of hCAP18/LL-37 (*CAMP*) in epithelial cells. The vitamin D binding protein (VDBP)-25(OH)D_3_ complex enters the epithelial cell from the circulation and 25(OH)${\text{D}}_{3}^{*}$ is subsequently released from the complex. In the cytoplasm, 25(OH)D_3_ is hydroxylated by 25-hydroxyvitamin D-1α-hydroxylase (CYP27B1; localized to the inner mitochondrial membrane) into the active metabolite 1,25(OH)_2_D_3_. 1,25(OH)_2_D_3_ subsequently binds to the nuclear vitamin D receptor (VDR) which heterodimerizes with the retinoic acid receptor (RXR) to interact with vitamin D response elements (VDREs) that are present on the promoter region of numerous genes, including *CAMP* (hCAP18/LL-37) and *CYP24A1* (25-hydroxyvitamin D-24-hydroxylase). 1,25(OH)_2_D_3_ thereby regulates its own negative feedback via direct induction of CYP24A1 that hydroxylates both 25(OH)D_3_ and 1,25(OH)_2_D_3_ either at the C-23 or at the C-24 to 23,25(OH)_2_D_3_ or 24,25(OH)_2_D_3_ and 1,23,25(OH)_3_D_3_ or 1,24,25(OH)_3_D_3_, respectively. These metabolites are further converted by CYP27B1 [that first converts 24,25(OH)_2_D_3_ into 1,24,25(OH)_3_D_3_] and CYP24A1 into 25(OH)D_3_-26,23-lactone and 1,25(OH)_2_D_3_-26,23-lactone or into calcitroic acid, metabolites that are excreted in the bile (50, 52–55). *The metabolism of 25(OH)D_3_ is shown in this figure, since there is more consensus regarding the metabolism of 25(OH)D_3_ and 1,25(OH)2D_3_ in literature.

Vitamin D enters the circulation either via food intake (plant-based: vitamin D_2_/animal-based: vitamin D_3_) or as a result of its synthesis in the skin by UVB radiation. It subsequently binds to the VDBP (30, 31), after which this complex is transported to the liver where it is converted by vitamin D-25-hydroxylases (CYP2RI and CYP27A1) into 25(OH)D. However, recent studies showed that also other cell types such as airway epithelial cells, keratinocytes, intestinal epithelial cells, and monocytes/macrophages express CYP2RI and CYP27A1, and thus are able to (locally) convert vitamin D_3_ into 25(OH)D_3_ (32, 33). This inactive 25(OH)D needs to be converted into the active 1,25(OH)_2_D by 25-hydroxyvitamin D-1α-hydroxylase (CYP27B1) in the kidney and in other cells, including several immune- and epithelial cells (34–40). 1,25(OH)_2_D regulates expression of several genes by binding the nuclear VDR, which heterodimerizes with the retinoic acid receptor (RXR) to interact with vitamin D response elements (VDREs) that are present on the promoter region of these genes (1, 2). VDR is most abundantly expressed in intestinal enterocytes, pancreatic islets, renal distal tubules and osteoblasts, but is also present at lower levels in most other tissues and several other epithelial- and immune cells (41–45). Expression of VDR is classically regulated by 1,25(OH)_2_D, growth factors and hormones such as FGF-23 and PTH, respectively, circulating calcium levels, bile acids, transcriptional co-activators/repressors, and genetic- and epigenetic modifications, which is tissue specific (46–49). 1,25(OH)_2_D regulates its own negative feedback by several mechanisms, including induction of expression of the catabolic enzymes 25-hydroxyvitamin D-24-hydroxylase (CYP24A1) and CYP3A4 (50, 51). CYP24A1 is expressed in most tissues and converts both 25(OH)D and 1,25(OH)_2_D into 23,25(OH)_2_D or 24,25(OH)_2_D and 1,23,25(OH)_3_D or 1,24,25(OH)_3_D, respectively (dependent on whether CYP24A1 hydroxylates at C-23 or at C-24). These are further converted into metabolites that have been found to be excreted into the bile (summarized in Figure 1) (50, 52, 56). CYP3A4 is mainly expressed in the liver and small intestines and contributes to the metabolic clearance of 25(OH)D and 1,25(OH)_2_D by converting 25(OH)D into 4β,25(OH)_2_D, and 1,25(OH)_2_D into 1,23R,25(OH)_2_D or 1,24S,25(OH)_2_D (51). Expression of both CYP27B1 and CYP24A1 in the kidneys is tightly regulated to maintain optimal Ca^2+^- and ${\text{PO}}_{4}{}^{2-}$ levels in the circulation, which are important for bone mineralization (57). In short, in response to low Ca^2+^ levels, parathyroid hormone (PTH) is secreted by the pituitary glands, which in turn reduces Ca^2+^ excretion and reabsorption of ${\text{PO}}_{4}{}^{2-}$ (57). PTH further induces expression of CYP27B1 and represses expression of CYP24A1 in the kidneys (57). This will increase the levels of 1,25(OH)_2_D in the circulation, which promotes intestinal Ca^2+^ and ${\text{PO}}_{4}{}^{2-}$ absorption (57). These elevated circulating Ca^2+^ and ${\text{PO}}_{4}{}^{2-}$ levels will subsequently induce expression of fibroblast growth factor 23 (FGF-23) in osteocytes and osteoblasts and impair secretion of parathyroid hormone (PTH) by the parathyroid glands (3). In the kidneys, FGF-23 suppresses expression of CYP27B1 and induces expression of CYP24A1, thereby inhibiting the synthesis and promoting degradation of 1,25(OH)_2_D (3). These complex mechanisms that explain how vitamin D and its metabolic enzymes maintain sufficient Ca^2+^ and ${\text{PO}}_{4}{}^{2-}$ levels in the circulation are more extensively discussed by Quarles et al. (57). In summary, it has become increasingly evident that the effects of vitamin D are not limited to homeostasis of Ca^2+^ and ${\text{PO}}_{4}{}^{2-}$ and bone mineralization, because several extra-renal cells such as airway epithelial cells and immune cells express the VDR and are capable of converting circulating 25(OH)D into the active 1,25(OH)_2_D metabolite.

## Mucosal Vitamin D Metabolism and Vitamin D Signaling in Chronic Inflammatory Airway Diseases

Local levels and activity of 1,25(OH)_2_D are in part determined by expression of VDR and the equilibrium between the vitamin D metabolic enzymes CYP27B1 and CYP24A1. It is important to realize that mucosal expression of CYP24A1, CYP27B1 and also VDR can be affected by several disease-associated inflammatory mediators, toxicants and pathogens, summarized in Table 1. As a consequence of this, the local availability of 1,25(OH)_2_D and/or VDR signaling in tissues such as the inflamed airways of patients that suffer from chronic inflammatory airway diseases might be reduced.

**Table 1:** Effects of inflammatory mediators on the expression of VDR, CYP24A1, and CYP27B1 in immune cells and epithelial cells.

**Cell/tissue type**	**Stimulus**	**Effect**	**References**
Primary airway epithelial cells	Poly(I:C); RSV; IL-13; IL-4; PM	CYP27B1 ↑	(38, 58–60)
	TNF-α; IL-1β; IL-17A; TGF-β1; NTHi	CYP24A1 ↑	(61, 62)
	CSE	CYP27B1 ↓	(63, 64)
	*A. fumigatus*; HRV; RSV	VDR ↓	(58, 65)
BEAS-2B (bronchial epithelial cell line)	HRV; RSV	VDR ↓	(58)
	PM	VDR ↑	(59)
16HBE (bronchial epithelial cell line)	*A. fumigatus*	VDR ↑	(21)
	TGF-β1	CYP27B1 ↑	(60)
	*A. fumigatus*	CYP27B1 ↑	(21)
A549 (lung carcinoma cell line)	CSE	VDR translocation ↓	(66)
HCT116 (colon cancer epithelial cell line)	LPS; TNF-α	CYP27B1 ↑	(67)
	LPS; TNF-α	VDR ↓	(67, 68)
	LPS	CYP24A1 ↓	(67)
COGA-1A (colon cancer epithelial cell line)	TNF-α ± IL-6	CYP27B1 ↓	(69)
Trophoblasts	TNF-α; IL-1β; IL-6	CYP24A1 ↑	(70)
	IFN-γ	CYP27B1 ↑	(70)
Macrophages	ss-RNA	CYP27B1 ↑ VDR ↑	(71)
Macrophages (derived from THP-1)	CSE	VDR ↑	(72)
Macrophages (derived from THP-1)	BaP	CYP24A1 ↑	(73)
Monocytes	TLR2/1L ± IFN-γ; LPS; IL-15	CYP27B1 ↑ VDR ↑	(39, 74–76)
	IL-4 ± TLR2/1L	CYP24A1 ↑	(39)
Neutrophils	IFN-γ *S. pneumoniae* T4R	CYP27B1 ↑ VDR ↑	(77)
T cells	T cell activators (anti-CD3/anti-CD28; PHA; PMA/ionomycin)	CYP27B1 ↑ VDR ↑	(78)
B cells	B cell activators (anti-IgM/anti-CD40/IL-21)	CYP27B1 ↑ VDR ↑	(79)

*Poly(I:C), Polyinosinic:polycytidylic acid; PM, Particulate matter; NTHi, nontypeable Haemophilus influenzae; A. fumigatus, Aspergillus fumigatus; CSE, Cigarette smoke extract; HRV, Human rhinovirus; RSV, Respiratory syncytial virus; ssRNA, Single stranded RNA; BaP, Benzo[a]pyrene; TLR2/1L, Toll like receptor 2/1 Ligand; PHA, Phytohemagglutinin; PMA, Phorbol 12-myristate 13-acetate*.

### Epithelial Cells

Chronic lung diseases are characterized by airway inflammation and impaired respiratory host defense, which is illustrated by the increased susceptibility for respiratory infections and exacerbations (25, 80, 81). Furthermore, exposure to inhaled toxicants such as cigarette smoke and air pollutants are associated with disease pathogenesis and exacerbations in COPD, CF and in asthma patients (82–84). It would therefore be of great interest to investigate these effects on local 1,25(OH)_2_D levels and on 1,25(OH)_2_D-mediated respiratory host defense in the airway mucosa. Studies in airway epithelial cells have shown that exposure to UV-inactivated non-typeable *Haemophilus influenzae* (NTHi) increased expression of the catabolic enzyme CYP24A1, whereas exposure to viral double stranded-RNA analog polyinosinic:polycytidylic acid (Poly[I:C]) increased expression of CYP27B1 and thereby conversion of 25(OH)D into 1,25(OH)_2_D, the active metabolite (38, 61). On the other hand, in the bronchial cell line BEAS-2B expression of VDR was decreased after infection with respiratory viruses such as human rhinovirus (HRV) and respiratory syncytial virus (RSV) (58). Collectively, these studies have shown in airway epithelial cells that respiratory viral- and bacterial infections can either promote or impair 1,25(OH)_2_D synthesis and responses.

A local airway inflammatory milieu can also exert differential effects on 1,25(OH)_2_D synthesis and signaling, dependent on the type of inflammatory mediators that are predominantly present. We have shown in differentiated primary airway epithelial cells that Th2 cytokines such as IL-4 and IL-13, enhance expression of CYP27B1 and expression of hCAP18/LL-37 upon 25(OH)D3 treatment, which suggests that a Th2-inflammatory environment, as found in allergic airway inflammation, increases the conversion of 25(OH)D into the active 1,25(OH)_2_D (83, 85). The observation that levels of both 1,25(OH)_2_D and hCAP18/LL-37 were increased in bronchoalveolar lavage (BAL) after allergen challenge is in line with this proposed mechanism (86). This effect of Th2 cytokines was in contrast to the effects (chronic) exposures to the proinflammatory cytokines IL-1β, TNF-α and IL-17A that strongly increased the expression of the 25(OH)D- and 1,25(OH)_2_D-degrading CYP24A1, even in absence of its inducer 1,25(OH)_2_D (61). Furthermore, short-term exposures to TGF-β1, a pleiotropic growth factor which is elevated in the lungs of COPD, CF and asthma patients, also increases the expression of CYP24A1 (62). As a consequence, 1,25(OH)_2_D-mediated expression of the AMP hCAP18/LL-37 was impaired, which was likely the result of the enhanced degradation of both 25(OH)D and 1,25(OH)_2_D by this enzyme (61, 62). In addition to pathogens and cytokines, exposure to inhaled toxicants such as cigarette smoke (CS) and particulate matter (PM) may also alter expression or activity of VDR and CYP27B1. Studies have demonstrated that cigarette smoking or exposure to CS extract (CSE) decreases expression of CYP27B1 and inhibited membrane bound (m)VDR translocation to the cell membrane in airway epithelial cells and A549 cells (an alveolar tumor cell line), respectively (63, 64, 66). This inhibition reduces the conversion of 25(OH)D to 1,25(OH)_2_D and 1,25(OH)_2_D-mediated gene expression as well as non-genomic actions of 1,25(OH)_2_D-membrane associated, rapid response steroid-binding (MARRS)- signaling (63, 64, 66). This adverse effect of cigarette smoking on the synthesis and effects of 1,25(OH)_2_D in airway epithelial cells was recently confirmed *in vivo* by Vargas Buonfiglio et al. who demonstrated that vitamin D supplementation increased antimicrobial activity in apical surface liquid (ASL) in the airway of healthy non-smokers, but not in smokers (64). On the other hand, exposure to PM increases the expression of both CYP27B1 and VDR in airway epithelial cells, thereby possibly promoting the synthesis and effects of 1,25(OH)_2_D (59). It is however important to consider that several retrospective and observational studies have demonstrated that air pollution is an independent risk factor for developing vitamin D deficiency (87). In conclusion, exposure to CS, TGF-β1 and presence of a proinflammatory milieu appeared to most strongly decrease local presence and signaling of 1,25(OH)_2_D in airway epithelial cells.

### Immune Cells

Whereas, various studies show that exposure to proinflammatory stimuli most likely affects local 25(OH)D and 1,25(OH)_2_D-levels and reduces the effects of 25(OH)D and 1,25(OH)_2_D in (airway) epithelial cells, the opposite appears to be the case for immune cells. In monocytes, macrophages and neutrophils, effects on 1,25(OH)_2_D synthesis and antimicrobial responses upon 25(OH)D treatment were generally enhanced by these proinflammatory stimuli as illustrated by increased expression of both VDR and CYP27B1 (39, 71, 74–77). It is therefore tempting to speculate that this apparent increase in antimicrobial responses upon 25(OH)D treatment in immune cells in an inflammatory environment may serve as a second line of defense and compensate for the enhanced epithelial degradation of 25(OH)D and 1,25(OH)_2_D during inflammation. Inhaled toxicants may also affect 1,25(OH)_2_D availability and responsiveness of immune cells. This is illustrated by two recent studies studying the effects of cigarette smoke on the human monocyte/macrophage-like cell line THP-1. One study showed that treatment with cigarette smoke extract (CSE) increased the expression of VDR without enhancing 1,25(OH)_2_D responses (72), while the other study -that focused on the effects of Benzo[a]pyrene (BaP) (a component produced by cigarette combustion)- demonstrated that 1,25(OH)_2_D -mediated CYP24A1 expression was induced, which was found to further enhance degradation of 1,25(OH)_2_D (73). In summary, proinflammatory stimuli generally increased the effect of 25(OH)D and 1,25(OH)_2_D on immune cells, whereas more studies are needed to fully determine the impact of exposure to cigarette smoke and other inhaled toxicants.

### Lung Mucosa

Whereas, these studies provide evidence that inflammation and inhaled toxicants may affect 25(OH)D and 1,25(OH)_2_D metabolism and responsiveness in epithelial cells and immune cells, it is not clear whether this has an impact on these events in lung tissue of patients with chronic lung diseases. Although evidence is limited, we can speculate that levels of 1,25(OH)_2_D and responses are also affected by disease-associated factors in mesenchymal cells that are present in the lung mucosa. One study that showed in a bleomycin fibrosis model and in primary lung mouse fibroblasts that TGF-β1 reduced expression of the VDR might support this assumption (88). It is currently insufficiently studied whether exposures to disease-associated factors promote or impair levels of 1,25(OH)_2_D and responses in immune-, mesenchymal and epithelial cells combined to give a better reflection of the *in vivo* situation. Interestingly, one study did already show that nasal CYP27B1- and 1,25(OH)_2_D-levels are both reduced in chronic rhinosinusitis (CRS) patients with nasal polyps as compared to CRS-patients without nasal polyps, whereas no difference was found in circulating 1,25(OH)_2_D-levels (89). Since most other studies were performed *in vitro* using monocultures of epithelial cells or immune cells, more complex models are needed to delineate this. Therefore, animal models or preferably more complex animal-free cell culture models using co-cultures or organs-on-chips models of primary fully differentiated epithelial cells, airway-derived fibroblasts or smooth muscle cells and immune cells could be considered in future studies.

## Protective Effects of Vitamin D on Mucosal Homeostasis

**Figure 2:**
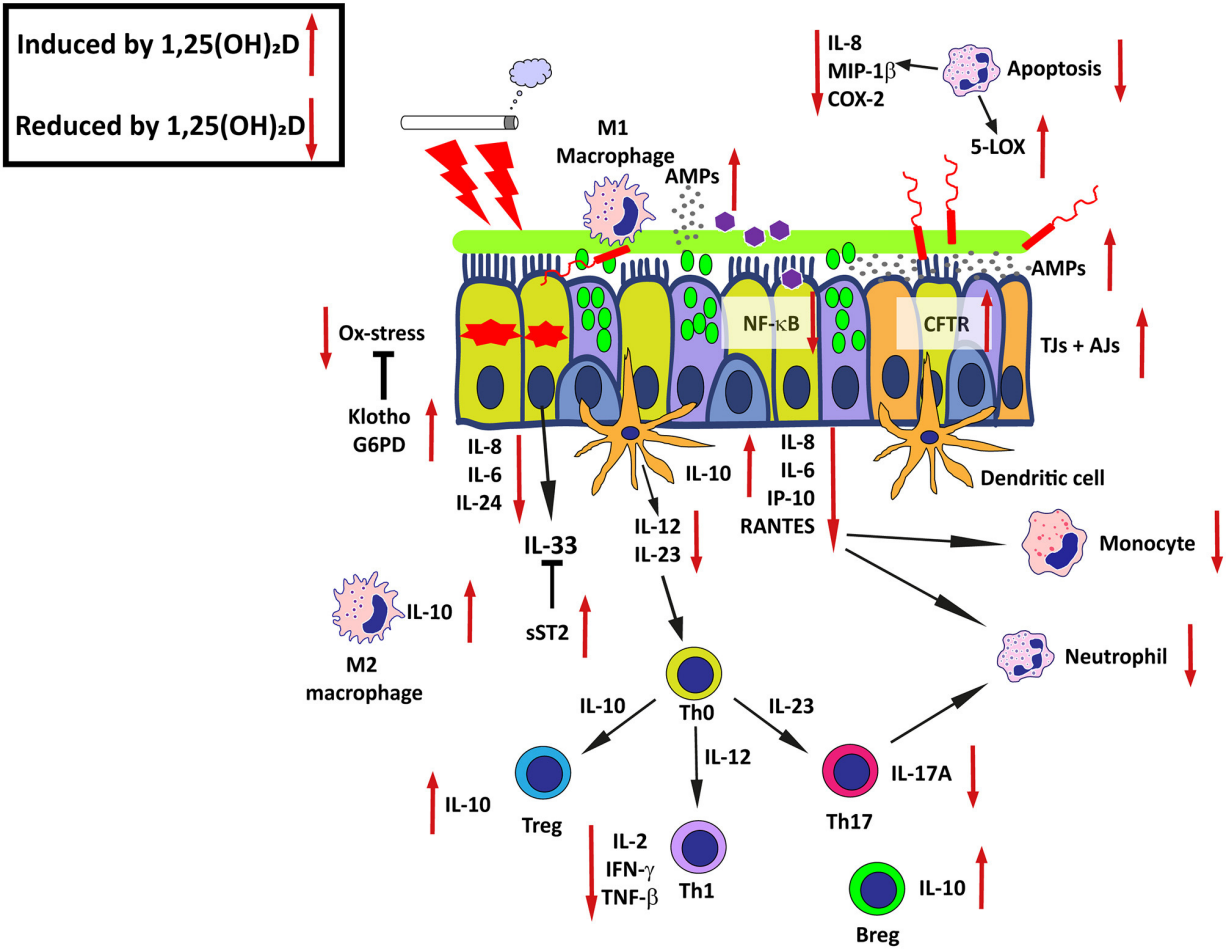
Effects of active 1,25(OH)_2_D on airway epithelial host defense-mechanisms in chronic airway disease. The promoting or inhibitory effects of 1,25(OH)_2_D are indicated by the red arrows. AMPs, Antimicrobial peptides; CFTR, Cystic fibrosis transmembrane conductance regulator; Ox-stress, Oxidative stress; SOCS, Suppressor of cytokine signaling proteins; TJs, Tight junctions; AJs, Adherens junctions; G6PD, Glucose-6-phosphate dehydrogenase; sST2, Soluble suppression of tumorigenicity 2; NF-κB, Nuclear factor kappa-light-chain-enhancer of activated B cells; Th0, Naieve T cell; Treg, Regulatory T cell; Th1, T helper type 1 cell; Th2, T helper type 2 cell; Th17, T helper type 17 cell; Breg, Regulatory B cell. See text for details and references.

After discussing altered 25(OH)D and 1,25(OH)_2_D metabolism and responsiveness in the inflamed airway mucosa, it is important to consider the possible consequences of these inflammation-induced changes in the airway mucosa keeping in mind the pleotropic effects of 1,25(OH)_2_D that were introduced earlier. In several cells, tissues and organs, 1,25(OH)_2_D regulates multiple cellular processes that affect normal and malignant cell growth and differentiation (90, 91). 1,25(OH)_2_D displays furthermore protective effects on mucosal host defense by maintaining the integrity of the epithelial barrier, inhibition of epithelial-to-mesenchymal transition (EMT), stimulating production of AMPs and modulating both innate- and adaptive immune functions (7, 29, 92). In addition, 1,25(OH)_2_D maintains both energetic and survival homeostasis in the mucosal epithelium through the modulation of stress and damage responses, including clearance of disturbing and stressful agents (3, 93) (Figure 2).

### Epithelial Barrier Function

In chronic inflammatory lung diseases, epithelial barrier function is impaired, and as a consequence the susceptibility toward respiratory infections is increased (94). There is increasing evidence that 1,25(OH)_2_D promotes epithelial barrier integrity or protects against epithelial barrier destruction. In cells of the bronchial epithelial cell line 16HBE, 1,25(OH)_2_D inhibited CSE-mediated reduction of the epithelial barrier and expression of E-cadherin and β-catenin (95). Recently, two murine studies were published that investigated the effects of vitamin D on pulmonary epithelial barrier function. Shi et al. showed that vitamin D-supplementation alleviated lung injury in LPS-treated mice through maintenance of the pulmonary barrier by inducing expression of Zonula occludens (ZO)-1 and occludin in whole lung homogenates (96), whereas Gorman et al. showed in healthy mice, fed with a vitamin D-poor diet, that vitamin D supplementation had little effect on epithelial integrity (97). Only the first study that used a more severe mouse model with higher levels of inflammation and edema found an effect of vitamin D on epithelial barrier function. Since inflammation is detrimental for epithelial barrier integrity (98), it cannot be excluded that the main protective effects of 1,25(OH)_2_D on the epithelial barrier in the first study by Shi et al. were in fact exerted through inhibition of inflammation rather than via direct induction of cell junction proteins. 1,25(OH)_2_D might also promote epithelial barrier function through its ability to increase expression of cystic fibrosis transmembrane conductance regulator (CFTR) in airway epithelial cells (32). CFTR maintains optimal ASL- and mucus hydration, volume and pH that support mucociliary clearance and activity of AMPs (99). Moreover, CFTR is also affected in the airways of smokers and COPD patients (100). In summary, these studies indicate that 1,25(OH)_2_D promotes both the integrity and function of the epithelial barrier and might additionally protect against epithelial damage by dampening inflammatory responses.

### Anti-fibrotic Effects of Vitamin D

The loss of epithelial barrier function with a decrease in epithelial polarization and cell-junction proteins and a gain of expression of mesenchymal markers is a hallmark of EMT (94). EMT is primarily involved in development, wound healing and stem cell differentiation, and TGF-β signaling plays a major role in this process (101). Elevated TGF-β1 levels are found in the lungs of patients with chronic inflammatory lung diseases and this was associated with cigarette smoking, inflammation and fibrosis (80, 102). There are indications that 1,25(OH)_2_D counteracts various pathways leading to EMT. In mouse models and in airway epithelial cell lines, vitamin D supplementation and 1,25(OH)_2_D, respectively, has been shown to inhibit EMT and fibrosis, in particular when this process is induced by TGF-β1 (88, 103–106).

### Effects of Vitamin D on Epithelial Antimicrobial Responses

In addition to maintenance of the epithelial barrier and inhibition of fibrosis as discussed in the previous paragraphs, vitamin D is also actively involved in respiratory host defense by a variety of mechanisms (3, 29). 1,25(OH)_2_D is an important inducer of AMPs, which are mostly cationic peptides that have a broad-spectrum antimicrobial activity, the ability to modulate immune responses and to promote epithelial wound repair and angiogenesis (107). hCAP18/LL-37 is likely to be the most prominent AMP that is induced by 1,25(OH)_2_D and is expressed in several types of mucosal epithelial cells and immune cells such as monocytes and neutrophils (38, 77, 108). In macrophages and intestinal epithelial cells, 1,25(OH)_2_D also increases expression of human β-defensin-2 (hBD-2), whereas in keratinocytes expression of both hBD-2 and human β-defensin-3 (hBD-3) is increased by 1,25(OH)_2_D (109–112). Collectively these data show that AMPs are modulated by 1,25(OH)_2_D in mucosal tissues, which could have impact on susceptibility to both bacterial and viral infections and on the composition of the microbiota, which will be discussed in the next section.

### Effects of Vitamin D on Innate and Adaptive Immune Responses

Diseases such as COPD and asthma are characterized by chronic inflammation, a low-grade and prolonged inflammation that may result in destruction and aberrant repair of surrounding tissue by growth factors, proteases and cytokines that are released at the site of inflammation (113–115). Cumulative data suggest that vitamin D exerts anti-inflammatory effects via its actions on both innate and adaptive immune responses. Upon viral infection or exposure of pro-inflammatory stimuli such as Poly(I:C) or PM, 1,25(OH)_2_D attenuates induced expression of cytokines and chemokines e.g., via inhibition of nuclear factor (NF)-κB or oxidative stress, respectively, in (airway) epithelial cells (38, 59, 116). Furthermore, 1,25(OH)_2_D increases expression of the soluble decoy receptor for IL-33 (sST2) by airway epithelial cells, which in turn inhibits the actions of the type 2 alarmin IL-33 (117). Further effects of 1,25(OH)_2_D on local innate and adaptive immune responses in the epithelial mucosa are mediated through its actions on immune and structural cells and have been reviewed by Heulens et al. (29) Vanherwegen et al. (118), and Pfeffer et al. (119).

Taken together, these findings suggest that on the one hand 1,25(OH)_2_D protects against infections by enhancing epithelial barrier function and production of AMPs, and on the other hand 1,25(OH)_2_D induces tolerance and dampens proinflammatory responses in various cell types of the airway mucosa. Thereby, 1,25(OH)_2_D may prevent exaggerated inflammatory responses and further damage to the mucosal tissue, qualities that are very relevant in the context of chronic inflammatory (lung) diseases (Figure 2).

### Effects of Vitamin D on Epithelial Oxidative Stress and Aging

COPD is considered to be a disease of accelerated aging lungs, underscored by markers of aging being increased in these patients partly as a result of oxidative stress (120). Evidence that 1,25(OH)_2_D may protect epithelial cells from oxidative stress was provided by Pfeffer et al. who demonstrated that 1,25(OH)_2_D increased expression of the antioxidant gene *G6PD* in airway epithelial cells. Furthermore, 1,25(OH)_2_D increased the ratio of reduced to oxidized glutathione and decreased the formation of 8-isoprostane after exposure to PM (59). The induction of klotho by 1,25(OH)_2_D might be another 1,25(OH)_2_D-mediated anti-aging mechanism (121). Klotho is an anti-aging protein that is mainly expressed in the kidney, brain and in the lung by airway epithelial cells and exerts its protective effects through the inhibition of inflammation, insulin/IGF-1 signaling and activation of forkhead transcription factor (FoxO) signaling, which enables removal of reactive oxygen species (ROS) (122–124). Expression of klotho is impaired in the airways of smokers and further decreased in the airways of COPD patients and in cultures of the bronchial epithelial cell line 16HBE after CSE exposure (124). These studies suggest that 1,25(OH)_2_D may protect against aging via inhibition of oxidative stress and possibly via its ability to restore klotho expression (Figure 2). However, direct evidence showing that 1,25(OH)_2_D indeed increases expression of klotho in airway epithelial cells is currently lacking.

### Effects of Vitamin D on Epithelial Autophagy and Apoptosis

In addition to providing protection against oxidative stress and aging, data from studies using intestinal epithelial cells suggest that 1,25(OH)_2_D may also promote cellular survival via the induction of autophagy and reduction of apoptosis (125, 126). In chronic inflammatory lung diseases, aberrant activation of autophagy plays a role in disease pathogenesis (127). A recent study showed that club cells and autophagy-related proteins were both decreased in COPD patients and that these proteins were important for club cell structure and function in airways (128). However, the effects of 1,25(OH)_2_D on autophagy in the airway mucosa of chronic inflammatory lung diseases are still unclear and need to be further evaluated (127).

## Role of Vitamin D in the Treatment of Chronic Airway Diseases

Clearly vitamin D has pivotal actions in host defense that are relevant in the context of chronic inflammatory lung diseases, in which vitamin D deficiency may be prevalent. Strategies to promote local levels of 1,25(OH)_2_D or use it as a treatment itself could be therefore of interest. Here, we will discuss the latest clinical evidence accompanied with functional *in vitro* and animal studies that may explain the effects of vitamin D supplementation on typical hallmarks of chronic airway diseases.

### Effect of Vitamin D on Inhaled Corticosteroid Responsiveness in Chronic Airway Diseases

Currently, inhaled corticosteroid (ICS)-use with or without long acting bronchodilators is the most frequently used treatment for COPD and asthma patients^1^. However, the response to corticosteroids is not always effective in many COPD patients and in patients with steroid resistant (SR)-asthma (129). There are several complex mechanisms that underlie the resistance to corticosteroids in both COPD and SR-asthma that include but are not limited to genetic background, impaired glucocorticoid receptor binding, T helper type 17 cell (Th17)-inflammation and oxidative stress (e.g., from air pollution or smoking) and decreased numbers of IL-10 secreting regulator T cells (Tregs), which normally prevent skewing toward Th17-inflammation (129). Direct evidence of the ability of 1,25(OH)_2_D to reverse SR was provided by a study showing that *ex-vivo* stimulation with 1,25(OH)_2_D promoted generation of IL-10–secreting Tregs which restored sensitivity toward corticosteroids in CD4+ T cells that were derived from SR-asthma patients (130). A further potential treatment role of 1,25(OH)_2_D was elegantly illustrated by studies that showed that vitamin D deficiency is associated with decreased steroid responsiveness in asthmatics and by the fact that several potential underlying mechanisms of SR such as oxidative stress and Th17-mediated inflammatory responses could be reversed by vitamin D treatment (59, 131–136). Interestingly, the corticosteroid dexamethasone was shown to increase expression of the 25(OH)D and 1,25(OH)_2_D degrading enzyme CYP24A1 in renal cells and osteoblasts (137), which suggests a bidirectional interaction between corticosteroids and 1,25(OH)_2_D and could further limit 1,25(OH)_2_D levels for patients. Additional research is needed to determine if vitamin D may also improve corticosteroid responsiveness in COPD.

### Vitamin D and Exacerbations in COPD

Exacerbations are a major burden for COPD patients, they accelerate decline in lung function and frequently result into hospital admissions (138, 139). Exacerbations are often triggered by pollutants or by bacterial- and/or viral infections (82, 140, 141). COPD patients generally have lower serum 25(OH)D levels than age- and smoking-matched controls, which is associated with more and more severe exacerbations (8, 10). Several *in vivo* and *in vitro* studies have provided evidence that explain the protective effects of vitamin D on exacerbations in COPD patients and this will be discussed accordingly.

#### Air Pollution

First of all, Pfeffer et al. showed that 25(OH)D and 1,25(OH)_2_D reduce the production of proinflammatory cytokines in part via the ability to enhance antioxidant responses in airway epithelial cells that were exposed to PM (59). This was also demonstrated in human DCs that were matured in presence of PM, where treatment with 1,25(OH)_2_D counteracted the expansion of proinflammatory IL-17A^+^ and IFN-γ^+^ Th17.1 cells (134). In line with this, Bolcas et al., showed that vitamin D supplementation counteracted the development of airway hyperresponsiveness and accumulation of Th2/Th17 cells in mice that had been repeatedly exposed to both diesel exhaust and house dust mite allergens (142). Vitamin D could therefore exert a protective role in air pollution-triggered exacerbations.

#### Respiratory Viral Infections

In addition to its protective effects against pollutants, there is also increasing evidence that 1,25(OH)_2_D may enhance clearance of respiratory viral infections that account for 30–50% as underlying cause of exacerbations in COPD patients (143). Infections with respiratory viruses such as HRV, coronaviruses and to a lesser extend respiratory syncytial virus (RSV) and (para)influenza virus are present during exacerbations and may predispose the host toward secondary bacterial infections that can eventually lead to uncontrolled bacterial outgrowth, more severe exacerbations and neutrophilic inflammation (143, 144). Two recent *in vitro* studies showed that acute exposure to relatively high doses (100–1000 nM) of 1,25(OH)_2_D reduced HRV-infection in undifferentiated cultures of airway epithelial cells (58, 145). In those models, 1,25(OH)_2_D most likely interfered with viral replication by increasing expression of interferon-stimulated genes and expression of hCAP18/LL-37, which has been shown to have direct antiviral activity (58, 145, 146). In fully differentiated airway epithelial cells, treatment with lower concentrations of 1,25(OH)_2_D (10 nM) during epithelial differentiation had no effect on acute HRV infection (147). As for other viruses than HRV, both Hansdottir et al. and Telcian et al. showed that 1,25(OH)_2_D did not decrease RSV infection in airway epithelial cells, but did reduce virus-induced inflammatory responses (58, 116). In addition, two other studies reported in influenza (H9N2 and H1N1)-infected A549 cells comparable findings (148, 149). Moreover, inhibitory effects of 1,25(OH)_2_D on poly(I:C)-induced inflammatory responses were furthermore confirmed in primary airway epithelial cells Hansdottir et al. and by our group (38, 85). Up to now, the afore mentioned studies suggest that higher doses of 1,25(OH)_2_D might be protective against HRV-infections in undifferentiated airway epithelial cells only, whereas for other respiratory viral infections 1,25(OH)_2_D mainly reduces inflammatory responses without affecting viral clearance. However, more studies are needed, especially in differentiated airway epithelial cells using multiple HRV-serotypes that use different receptors for infection to verify if 1,25(OH)_2_D indeed is capable of promoting HRV-clearance. There is more consensus about 1,25(OH)_2_D reducing virus-induced inflammatory responses and this may certainly help to alleviate the burden of exacerbations in COPD (38, 85).

#### Bacterial Infections

In addition to viral infections, also bacterial infections are associated with COPD exacerbations and account for ~50% of all exacerbations (150). Due to improved study design and sampling techniques from the lower airways using bronchoscopy in recent decades, the causative role of bacteria in COPD-related exacerbations has become clear (150). This was additionally supported by Sethi et al., who found that acquisition of a new strain of pathogenic bacterial species into the airways was linked to COPD exacerbations (151). Recent developments in assessing the airway microbiota using 16S rRNA sequencing techniques further demonstrated that during exacerbations, the relative abundance of Haemophilus, Pseudomonas, and Moraxella was increased and the microbial composition was shifted toward the Proteobacteria phylum (141). The ability of 1,25(OH)_2_D to promote antibacterial activity was recently demonstrated in cultures of airway epithelial cells. In differentiated airway epithelial cells, we have shown that both 25(OH)D and 1,25(OH)_2_D treatment enhances epithelial expression of hCAP18/LL-37 and antibacterial activity against NTHi, a Gram-negative bacterium, which is associated with COPD exacerbations (61, 152). In addition, Yim et al. demonstrated that 1,25(OH)_2_D treatment increased expression of the AMP hCAP18/LL-37 and killing of *Pseudomonas aeruginosa* and *Bordetella bronchiseptica*, which are both Gram-negative bacteria (153). These observed antibacterial effects of 1,25(OH)_2_D on airway epithelium *in vitro* were recently confirmed *in vivo* by Vargas Buonfiglio et al. The authors demonstrated that vitamin D supplementation increased antimicrobial activity against the Gram-positive *Staphylococcus aureus* in ASL in healthy non-smokers and was dependent on presence of hCAP18/LL-37 (64).

In murine airways, studies showed no effects of 1,25(OH)_2_D on the expression of *Defb4* or *mCramp* (the murine homolog for *CAMP*) (154). This can be explained by the fact that both the promotors of *mCramp* and *Defb4* lack VDREs, suggesting that mice might not be suitable for studying the role of 1,25(OH)_2_D in AMP-mediated host defense in infection (155). Indeed, Niederstrasser et al. showed no effects of vitamin D deficiency on the susceptibility of mice to pulmonary infection with *Streptococcus pneumoniae* or *Pseudomonas aeruginosa* (156). However, in a recently developed mouse model by Lowry et al., who transfected *mCramp* knockout mice with the human *CAMP* gene, topical vitamin D_3_ treatment increased expression of *CAMP* and promoted antibacterial effects on the mucosa of the skin (157). There are also multiple other murine studies that demonstrate protective effects of vitamin D on bacterial infections in the gut, indicating that 1,25(OH)_2_D -mediated antibacterial effects are additional modulated by other mechanisms such as via enhancement of epithelial barrier integrity (67, 158). In conclusion, these observations show that 1,25(OH)_2_D promotes protection against pollutants and enhances clearance of viral– and bacterial infections (both Gram-positive and negative bacteria) in combination with a dampening effect on exaggerated immune responses and these features might explain why vitamin D (deficiency) is linked to COPD exacerbations.

### Modulation of Microbiota by Vitamin D

There are strong indications that modulation of immune responses and antibacterial activities by 1,25(OH)_2_D and/or 1,25(OH)_2_D-regulated AMPs as well as autophagy have implications for the composition of the microbiota at the epithelial mucosa of the airways and the gut (159). Evidence for a role of AMPs in regulating the composition of the microbiota in the gut came from a variety of studies, including those showing that Paneth cell-derived defensins may modulate the composition of the microbiome (160). This notion is further supported by observations showing that many commensal gut bacteria are protected from killing by AMPs such as the 1,25(OH)_2_D-inducible hCAP18/LL-37 and hBD-2, whereas pathogens are in general more sensitive (161). Alterations in the gut microbiota have been linked to many diseases of the gut such as IBD but also with diseases affecting the lungs such as COPD and asthma, implicating an important role for the so-called gut–lung axis (162, 163). The mechanisms that explain how gut microbiota affect lung health and disease are complex and include the production of short chain fatty acids (SCFAs). SCFA have a wide range of effects on both immune and structural cells, and the effect of SCFA produced in the intestine on lung immunity may in part be explained by modulation of myeloid cells in the bone marrow, which subsequently migrate to the airways and modulate local immune responses (163). Microbiota that are diverse, rich and contain a higher abundance of SCFA-producing species within these populations are considered to be associated with health (164). In the gut there is strong evidence that both vitamin D deficiency and/or supplementation affect composition of the adult and infant microbiota (164, 165), specifically in relation to disease (166). However, due to the limited number of RCTs and small sample sizes, the precise effects on the microbiota and the mechanisms involved in this are still unclear (164). Alterations in the lung microbiota are also observed in COPD and asthma patients and are likely the result of environmental exposures, airway remodeling, infections and treatments such as the use of antibiotics. This may contribute to disease pathogenesis through altered epithelial innate and adaptive immune responses that damages the airway epithelial barrier and provokes further changes in the lung microbiome that accumulates with increasing disease severity (167, 168). To date only 2 studies describe a possible influence of vitamin D on composition of the microbiota in the airways (169, 170). Toivonen et al. showed an association between low serum 25(OH)D levels and reduced richness of the nasopharyngeal microbiota and bronchiolitis severity in patients with low 25(OH)D levels (169), whereas in another study vitamin D supplementation decreased the abundance of *Staphylococcus aureus, Staphylococcus epidermidis* and Corynebacterium species in sputum samples in vitamin D-deficient CF patients compared to sufficient CF patients (170). In summary, there is evidence that alterations in the airway or gut microbiota can affect chronic airway disease and that these changes could be related to both vitamin D deficiency and/or supplementation. However, due to the limited number of RCTs and small sample sizes more RCTs are needed in larger patient populations.

### Effect of Vitamin D Supplementation on Chronic Airway Diseases

#### COPD

The above described protective and therapeutic possibilities of vitamin D, together with observations that many COPD patients are vitamin D deficient, suggest that COPD patients might benefit from vitamin D supplementation. As discussed elsewhere in this review, the link between circulating 25(OH)D levels and the number of exacerbations has been extensively studied (8). So far however, only 4 RCTs have investigated the effect of vitamin D supplementation in the context of COPD: only 2 out of 4 RCTs showed that vitamin D supplementation reduces the number of exacerbations (171–174). However, in a *post-hoc* analysis, selecting those patients that were vitamin D deficient, exacerbations were indeed reduced after vitamin D supplementation. Jolliffe et al. summarized these 4 RCTs and performed a recent individual participant data meta-analysis and concluded that vitamin D supplementation is only protective against exacerbations in COPD patients with baseline serum 25(OH)D levels <25 nmol/L (175). These important findings suggest that exacerbations in this specific subset of COPD patients are connected to vitamin D deficiency and this part can be resolved with supplementation. In summary, the protective effects of vitamin D in patients suffering from COPD are most prominent in those with vitamin D deficiency and this would indicate that serum levels 25(OH)D in these patients should always be determined before considering using vitamin D supplementation. Since only 4 RCTs with relatively small patient populations have been conducted in both vitamin D-sufficient and -deficient COPD patients, more RCTs are needed, especially in vitamin D-deficient patients. Currently, a multicenter RCT is being conducted by Rafiq et al. in a group of vitamin-deficient COPD patients (25(OH)D <50 nmol/L), which may reveal whether vitamin D supplementation is indeed protective against exacerbations in this group (176).

#### Vitamin D Supplementation in Asthma, Cystic Fibrosis and Acute Respiratory Tract Infections

In addition to the effects of vitamin D supplementation in COPD patients, the effects of vitamin D supplementation has also been extensively investigated in other lung diseases (which have associations with vitamin D deficiency) such as asthma, cystic fibrosis, upper respiratory tract infections. Most RCTs that investigated the effects of vitamin D supplementation were performed in acute respiratory tract infections (ARTIs) and asthma. A recent meta-analysis that assessed the effects of vitamin D supplementation in 25 RCTs (11,321 participants) showed that indeed vitamin D supplementation was protective against ATRIs and this effect was again more profound in patients with vitamin D deficiency 25(OH)D <25 nmol/L at baseline (177). A recent meta-analysis in asthma that included a total of 14 randomized controlled trials (1,421 participants), indicated that vitamin D supplementation reduced the rate of asthma exacerbations and increased lung function, especially in patients with vitamin D insufficiency (25(OH)D <75 nmol/L) (178). Interestingly, in asthma patients that were supplemented with vitamin D, the frequency of respiratory infections was reduced, and this effect was related to the increase of hCAP18/LL-37 (179). CF patients with vitamin D deficiency had a higher rate of exacerbations as compared to patients with sufficient 25(OH)D levels (180). However, only one recent multicenter RCT was conducted and indicated that vitamin D supplementation did not affect the number of exacerbations in CF patients with serum 25(OH)D concentrations between 25 and 137.5 nmol/L (181). In summary, the protective effects of vitamin D supplementation in patients suffering from COPD, asthma or ARTIs are most prominent in those with vitamin D deficiency and this would indicate the importance of establishing serum levels 25(OH)D in these patients as supplementation could reduce unnecessary aggravated disease pathology as a result of this deficiency.

## Conclusion and Perspectives

Many drivers of COPD pathogenesis such as chronic exposure to noxious particles and gases, which are present in CS and air pollution, proteolytic enzymes, cytokines and chemokines that are released by infiltrating inflammatory cells, are known to harm the epithelial barrier and cause aberrant remodeling of the airway epithelium with important functional consequences for e.g., host defense. A dysfunctional epithelial barrier increases the susceptibility toward bacterial and viral infections, which are important triggers of COPD exacerbations and these exacerbations contribute importantly to disease progression. Sufficient local levels of 1,25(OH)_2_D may provide partial protection against these effects by reducing the effects of oxidative stress induced by exposure to inhaled oxidants or those derived from recruited inflammatory cells. 1,25(OH)_2_D furthermore protects against impairment of epithelial barrier function by promoting the integrity of the epithelial barrier, and by modulating both innate and adaptive immune responses. Protection against the detrimental effects of both bacterial and viral infections is provided by the ability of 1,25(OH)_2_D to promote of antiviral responses, induce expression of AMPs and modulate of inflammatory responses. Taken together, these activities suggest that 1,25(OH)_2_D may provide protection against development and progression of COPD, and against disease exacerbations.

In addition, the local inflammatory milieu as well as the chronic exposure to noxious particles and gases, which are present in CS and air pollution, may negatively affect synthesis and signaling of 1,25(OH)_2_D. Here we discussed recent *in vitro* studies that demonstrated that disease-associated factors such as inflammation and exposure to CS and air pollution could interfere with 1,25(OH)_2_D signaling and its degradation and activation by affecting expression of VDR, CYP24A1 and CYP27B1, respectively. These findings indicate that 1,25(OH)_2_D levels and its activities on airway mucosa might be impaired especially in patients with COPD with exposures to cigarette smoke and cytokines such as TNF-α, IL-1β, IL-17A and TGF-β1. This suggests that even in patients with sufficient 25(OH)D serum levels the local activity of 1,25(OH)_2_D in the lungs can be improved. We have to start generating more information on both systemic and local 1,25(OH)_2_D levels and gene expression signatures related to 25(OH)D and 1,25(OH)_2_D metabolism or responses in COPD (and other chronic inflammatory diseases that are related to vitamin D deficiency), both at baseline and after vitamin D supplementation. This information could lead to improved treatment strategies that enhance local efficacy of 1,25(OH)_2_D, using e.g., specific CYP24A1-inhibitors such as VID400 (182). Alternatively, degradation by CYP24A1 could be prevented by using 1,25(OH)_2_D analogs that are insensitive to CYP24A1-mediated degradation, such as sulfone and sulfoximine derivatives, that also act as a VDR agonist (183). A third option is to entail the use of combination treatment with vitamin D and anti-inflammatory or certain anti-fibrotic drugs that target cytokines/proteins that are known to potentially decrease local levels and signaling of 1,25(OH)_2_D by inducing expression of CYP24A1 (48, 184, 185). When considering such strategies, it should be noted that these may enhance the calcemic side effects and lead to unwanted inhibition of the immune system. We therefore need to carefully analyze the preclinical *in vivo* and *in vitro* studies and balance the pros and cons of the different strategies. In conclusion, future studies in COPD and but also in other chronic inflammatory diseases that are related to vitamin D deficiency, should be designed with more focus on assessing and improving local levels of 1,25(OH)_2_D. These new insights may lead to the development of new treatment strategies, such as those targeting CYP24A1 to enhance local 1,25(OH)_2_D resulting in improved homeostasis and protection of the airway mucosa in patients with chronic inflammatory lung diseases.

## Author Contributions

JS, AD, and PH: Conception and design. JS: Analyzing literature and drafting the manuscript. AD and PH: Revision of the manuscript. All authors: reviewed the manuscript and agree with its submission.

## Conflict of Interest

The authors declare that the research was conducted in the absence of any commercial or financial relationships that could be construed as a potential conflict of interest.

## Footnotes

^1^https://goldcopd.org (2019).
